# In-silico Design of DNA Oligonucleotides: Challenges and Approaches

**DOI:** 10.1016/j.csbj.2019.07.008

**Published:** 2019-07-29

**Authors:** Michaela Hendling, Ivan Barišić

**Affiliations:** Austrian Institute of Technology GmbH, Center for Health & Bioresources, Molecular Diagnostics, Giefinggasse 4, 1210 Vienna, Austria

**Keywords:** Oligonucleotide design, Sequence database, Alignment, Oligonucleotide secondary structures

## Abstract

DNA oligonucleotides are essential components of a high number of technologies in molecular biology. The key event of each oligonucleotide-based assay is the specific binding between oligonucleotides and their target DNA. However, single-stranded DNA molecules also tend to bind to unintended targets or themselves. The probability of such unspecific binding increases with the complexity of an assay. Therefore, accurate data management and design workflows are necessary to optimize the *in-silico* design of primers and probes. Important considerations concerning computational infrastructure and run time need to be made for both data management and the design process. Data retrieval, data updates, storage, filtering and analysis are the main parts of a sequence data management system. Each part needs to be well-implemented as the resulting sequences form the basis for the oligonucleotide design. Important key features, such as the oligonucleotide length, melting temperature, secondary structures and primer dimer formation, as well as the specificity, should be considered for the *in-silico* selection of oligonucleotides. The development of an efficient oligonucleotide design workflow demands the right balance between the precision of the applied computer models, the general expenditure of time, and computational workload.

This paper gives an overview of important parameters during the design process, starting from the data retrieval, up to the design parameters for optimized oligonucleotide design.

## Graphical Abstract

**Figure:**
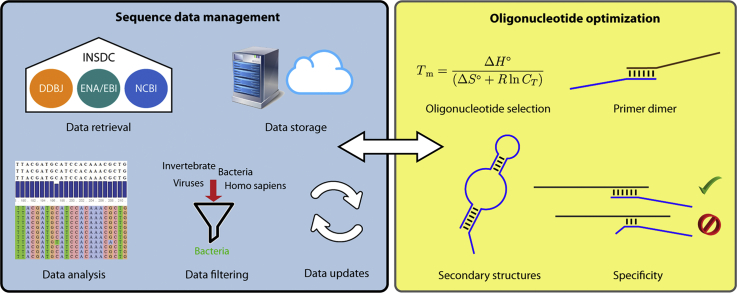

## 1 Introduction

**Fig. 1:**
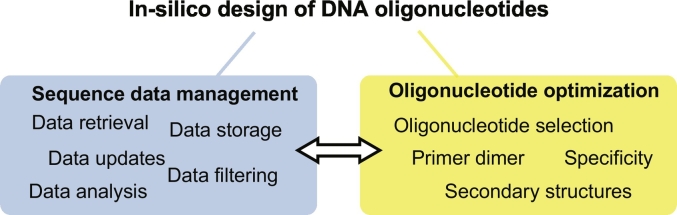
Essential parts for the *in-silico* design of oligonucleotides. The design process is divided into two parts. First the sequence management, comprising data retrieval, updates, storage, filtering and analysis. Second the actual optimization of the oligonucleotides, involving the selection and specificity, as well as checks concerning primer dimer and secondary structure formation.

DNA oligonucleotides are used in research, diagnostics and therapeutics, for example as primers and probes. Ranging from polymerase chain reaction (PCR) to microarrays and other hybridization-based reactions, technologies involving oligonucleotides proved themselves as cost-effective routine applications in the field of molecular biology and biotechnology. The selection of primers and probes is a critical component for the overall success of oligonucleotide-based experiments. Although, selecting single oligonucleotides can be performed manually without much computational effort, constructing multiplex assays involving multiple primer sets becomes more challenging [1]. Here, accurate sequence data management and design of primers and probes are essential for successful reactions (Fig. 1). First, a comprehensive and up to date database comprising both target and non-target DNA is necessary for the efficient design of specific oligonucleotides. The target DNA forms the basis for the creation of oligonucleotides, whereas non-targets are used to prevent the design of unspecific oligonucleotides. Therefore, high-quality sequences are essential to perform oligonucleotide selections. Second, it is important to use a design workflow that accurately simulates the experimental behavior of primers and probes prior to the in-vitro evaluations, to optimize the use of time and lab resources. A high number of design applications were developed in the last decades, improving the state of the art *in-silico* oligonucleotide design [2]. Additionally, computational infrastructure and maximum run time, which can form potential limitations, have to be taken into account for both data management and the design process.

This publication gives an overview of the essential processes, for accurate *in-silico* probe and primer design involving comprehensive background data sets. Starting with a short introduction of the different applications of oligonucleotides, it discusses important considerations during the design process, from the data retrieval, up to design parameters. The aim of this paper is to summarize common challenges and possible approaches concerning oligonucleotide design for researchers who want to set up their own design pipelines without giving detailed information on experimental procedures. For readers who are interested in a more detailed review targeting experimental approaches in combination with software tools for oligonucleotide design, we recommend the review by Noguera et al. [3].

### 1.1 Applications of Oligonucleotides

Oligonucleotides are specifically designated for their different applications, and thus, they differ in their length, composition and other critical parameters. The following subsections shortly introduce the most common applications of oligonucleotides.

#### 1.1.1 PCR

PCR is the most common application in which oligonucleotides are used as primers. Since its introduction in 1985, PCR has become a fundamental technique in molecular biology [4]. It is a nucleic acid amplification method that enables the generation of large amounts of newly synthesized DNA derived from significantly smaller amounts of template DNA. PCR basically consists of three main steps including denaturation, annealing and extension. The denaturation step separates the double-stranded template DNA into single strands under heat exposure. The denaturation is necessary to enable the subsequent annealing step where pairs of inwardly facing primers bind to the single-stranded template DNA. Finally, primers are elongated by a DNA polymerase during the extension step. The polymerase adds nucleotides to the primers and generates multiple copies of the intervening target DNA regions [5].

Real-time (RT-PCR) or quantitative PCR (qPCR) is an established method for the quantification of DNA. TaqMan probes and molecular beacons are popular sequence-specific probes that are used in qPCR to increase the specificity of the detection reaction. The TaqMan probe is a sequence-specific oligonucleotide that consists of a reporter fluorescent dye at its 5′ end and a quencher dye at its 3′ end. During the primer extension, the TaqMan probe is hydrolyzed by the 5′ to 3′ exonuclease of the polymerase, leading to the separation and finally to the release of the fluorophore. A fluorescent light signal, whose intensity is proportional to the number of PCR products, is detected by the RT-PCR instrument. If the probe is not hydrolyzed by the DNA polymerase, the quencher dye will absorb the fluorescent light emitted by the reporter dye [6]. Molecular beacons are oligonucleotide probes that have a stem-loop structure. The stem consists of two complementary sequences, and the loop is complementary to the target sequence. As soon as the probe binds to the target DNA, the fluorophore attached to the 5′ end of the stem separates from the quencher that is attached to the other end of the stem and a fluorescent signal is emitted [7].

#### 1.1.2 Microarrays

DNA microarrays or DNA chips are used for the detection of multiple genes, species, genotyping viruses, testing multiple single nucleotide polymorphisms (SNPs), or performing gene expression studies [8]. For this, oligonucleotides are attached to a solid surface. The detection reaction is based on a hybridization reaction of labeled target DNA to specific spots [9]. The hybridization is usually detected and measured by a fluorescent signal and its total intensity depends on the amount of target sample bound to the spot. However, alternative detection principles, such as enzymatic, electrochemical and plasmonic, were developed in the past decades [[10], [11], [12]].

#### 1.1.3 Fluorescence In Situ Hybridization

Fluorescence In Situ Hybridization (FISH) is used to detect and visualize DNA and RNA in tissues or cells. Target cells are fixed to conventional slides and permeabilized to allow target accessibility. During hybridization, specific probes that are tagged with fluorophores or alternative labels, bind to complementary nucleic acids. After removing excess oligonucleotides during a washing step, the results can be visualized using fluorescence microscopy or other imaging systems [13].

#### 1.1.4 Antisense Oligonucleotides

A technology for nucleic-acid-based gene silencing and modification was introduced with antisense oligonucleotides (ASOs). These nucleic acid sequences are chemically modified oligonucleotides that can regulate the translation of genetic material into functional proteins. The suppression of translation is achieved by binding of ASOs to complementary mRNA [14]. The formation of a duplex structure upon binding of the oligonucleotide to the mRNA prevents the correct processing of the mRNA, and thus, the translation of the encoded protein. Therefore, ASOs are effective tools for basic molecular biology, proteomics research, drug discovery, and genomics.

## 2 Data Management

**Fig. 2:**
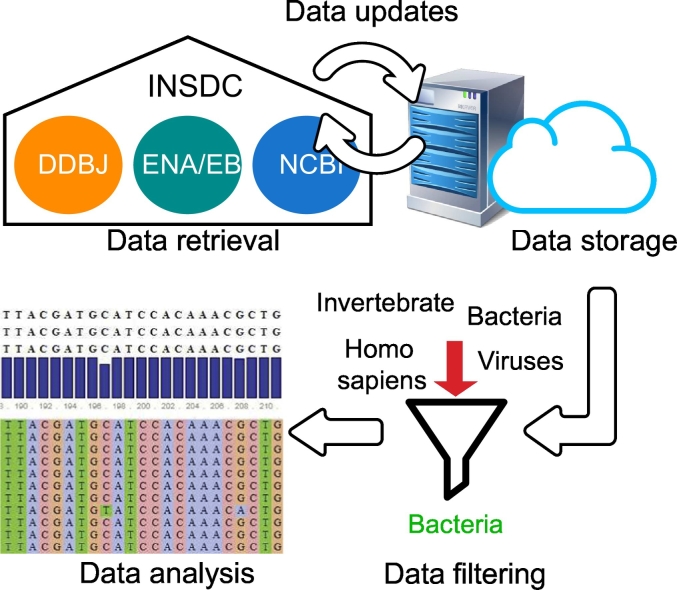
Illustration of a possible data management workflow for *in-silico* oligonucleotide design.

Any application that utilizes oligonucleotides should be based on a well-defined set of sequence data, comprising targets that should be amplified or detected, as well as sequences that should not be targeted by the designed oligonucleotides. Proper data management covering data sources, retrieval, filtering and analysis is necessary to provide an adequate data basis for oligonucleotide design (Fig. 2). Upon the identification of the optimal sequence data source, it is even more important and challenging to define the data retrieval and storage procedure. Users need to be able to efficiently analyze the retrieved data and design oligonucleotides. This section gives an overview of sequence data management for oligonucleotide design. It discusses hardware and software infrastructure required to perform efficient sequence data management, including data maintenance and curation. In many cases, it is useful to filter sequences to increase the efficiency of the *in-silico* design. The retrieved data sets for oligonucleotide design usually require further analysis before they are used for primer and probe selections. Adequate tools that are compatible with the data management system need to be selected to prepare the sequence data for the actual *in-silico* oligonucleotide design. Based on where the sequence data management system is hosted, users can choose between web services or local installations. The following subsections address data retrieval, filtering and analysis using web services and local software including the common limitations.

### 2.1 Data Sources

**Fig. 3:**
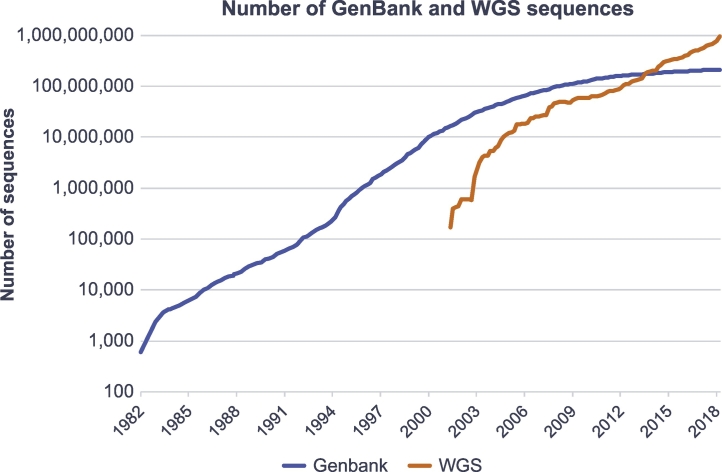
Number of sequence records in each release of GenBank from 1982 till 2019 and of WGS data from 2002 till 2019. The data has been retrieved from https://www.ncbi.nlm.nih.gov/genbank/statistics/.

A comprehensive resource for biological data is provided by the International Nucleotide Sequence Database Collaboration (INSDC [15]). This initiative operates between the National Center for Biotechnology Information (NCBI [16]), the DNA Data Bank of Japan (DDBJ [17]), and the European Molecular Biology Laboratory's European Bioinformatics Institute (EMBL-EBI [18]). These databases give researchers the possibility to freely access, download, and analyze sequence data. DDBJ is a public database of nucleotide sequences including web services for data submission, retrieval, a Web API, and a DDBJ Read Annotation Pipeline [19]. NCBI offers a large suite of online resources covering literature, health, genomes, genes, proteins, and chemicals [20]. EMBL-EBI provides free access to biological data and literature comprising DNA and protein sequences, biomolecules and their characteristics as well as tools for data analysis and discovery [21]. All three databases provide access to comprehensive data sets and services for data download via web service, programmatic access and the File Transfer Protocol (FTP). For example, the European Nucleotide Archive (ENA [22]), which is part of EMBL-EBI, currently stores over two billion sequences. Also, the databases of NCBI, such as GenBank and the whole genome sequence (WGS) database, comprise millions of sequences (Fig. 3). The increase of WGS data from 100,000 to over a billion sequences within approximately 20 years, reflects the tremendous growth in biological data.

Besides these large and general public data repositories, more specialized databases can also serve as helpful sources for oligonucleotide design. The SILVA ribosomal RNA gene database, for example, provides a comprehensive web resource for up to date and quality-controlled ribosomal RNA sequences [23]. Another example is the Comprehensive Antibiotic Resistance Database [24]. This manually curated database holds detailed information on antimicrobial resistance regarding the involved genes, proteins, and mutations.

### 2.2 Filtering

Filtering means the reduction of the number of sequences in the database and simultaneously an increase of the target sequence quality. An increase of the sequence database quality means that appropriately applied filtering can lead to the exclusion of sequences which are not necessary for the primer and probe design. Filtering can include taxonomic ranks, such as only sequences from a specific species within the data set. Another possibility would be to filter by sequence characteristics, such as the presence of a start and stop codon within the target sequences.

### 2.3 Analysis

One very efficient way to analyze sequences, before designing oligonucleotides, is via sequence alignments, which enable the organization and visualization of nucleotide positions based on similarity. Pairwise alignments involve only two sequences, whereas multiple sequence alignments align more than two sequences. A distinction is made between two methods, local and global alignments. Local alignments generate alignments of the most similar regions, whereas global alignments align all input sequences [25,26]. Additionally, EMBL-EBI provides genomic alignments which focus on characteristics present in genomic data. Alignment tools are useful for oligonucleotide design, as they can be used to create groups of homologous sequences, check whether a target gene contains specific single nucleotide polymorphisms (SNPs), get information concerning functional, evolutionary, or structural relationships or to find possible sequencing errors. The most common freely available multiple sequence alignment tools for nucleotide sequences are shown in Table 1.

**Table 1:** Common multiple sequence alignment tools for nucleotide sequences.

Name	Method
Clustal omega [27]	Progressive alignment, guide tree
Kalign [28]	Progressive alignment, Wu-Manber string-matching algorithm
MUSCLE [29]	Progressive alignment, iterations
MAFFT [30]	Progressive alignment, iterations
T-Coffee [31]	Progressive alignment
DIALIGN [32]	Greedy and progressive alignment

### 2.4 Web Services

#### 2.4.1 Data Retrieval & Filtering

Several web services provided by the online data repositories can be used to manage and analyze sequences without downloading any data. For example, NCBI provides a service where users can query the database using a search key (e.g. gene name or accession number), save the results to a private collection, and eventually analyze the data within this collection using the online Basic Local Alignment Search Tool (BLAST) [33]. Search results from EBI Search can also be directly submitted to their online implementation of Clustal Omega for sequence alignment. Filtering can be applied directly via the web sites of the data repositories. For example BLAST, NCBI GenBank, and the EBI Search engine of EMBL-EBI provide possibilities to filter data directly upon retrieval [34,35]. NCBI GenBank also allows users to control the quality by means of restricting the sequence retrieval to curated data sets, such as the RefSeq database [36].

NCBI includes the option to directly pick primers from single sequence records using Primer-BLAST [37]. However, this option is often not acceptable, as it does not include preceding sequence data analysis. Further information on primer design using Primer-BLAST is discussed in section 3 Oligonucleotide design.

#### 2.4.2 Analysis

The analysis can be accomplished without the need to download any sequence data and analysis tools. For example, BLAST provides a fast way to find regions of similarity between biological sequences. The advantage of the online version of BLAST is that target sequences can be aligned to comprehensive online databases. A high number of other sequence alignment tools that have similar functions such as Clustal Omega, MUSCLE, MAFFT, and T-Coffee, are available online (see Table 1). Besides the visualization of sequence similarity, such alignment tools can be used to form groups of similar sequences. A more direct approach to form user-defined sequence similarity groups is provided by Cd-hit and UCLUST [38,39]. These tools also calculate alignments on a set of input sequences, but their main task is to create sequence clusters. Both tools are very fast, but the results presented to the users only comprise the sequence clusters and no alignments. We experienced that it is often advisable to visually examine the alignments of sequences before forming sequence groups, for example, if erroneous sequences are in the data set (e.g. sequencing errors or annotation errors). The USC Genome Browser provides a web interface to a comprehensive set of tools to access and analyze genome data [40]. It can be used to map sequences to a specific genome displaying certain features within the alignment. This feature is useful if specific regions within a genome, like specific genes or coding sequences, need to be visualized and selected for oligonucleotide design. Due to its wide variety of analysis and display options, it is advisable to do some initial training and tutorials before using this tool. The same applies to the Galaxy platform, a web-based framework for data-intensive biomedical analyzes [41]. This platform provides a wide range of tools to perform alignments, assemblies, annotations, visualizations, statistical calculations, etc.

#### 2.4.3 Limitations

Data retrieval via online portals of data repositories often shows limitations concerning the number of queries at a time, query size and usage of background databases. Furthermore, the user is limited to the tools provided by the web service. Using the search engines of NCBI, DDBJ or EMBL-EBI can be also very impractical if someone has to perform hundreds of queries to build larger data sets. Sequence analysis often requires alignments and visualization of thousands of sequences at once, but the provided online tools for data analysis are limited in the number of input sequences. The web service of Clustal Omega provided by EMBL-EBI can align a maximum number of 4000 sequences. Though the online BLAST engine provides updated background databases for the alignment, only one database from a predefined set of databases can be selected at a time. Its maximum number of aligned sequences to display is limited to 20,000. In some cases, this might not be enough.

Another non-negligible bottleneck of web data retrieval and analysis tools is that most of these tools are independent applications. Users need to shift data from one service to another and start each tool separately. Such workflows are very inefficient and error-prone, especially, if a lot of data needs to be analyzed. Givan et al. published their Personal Sequence Database (PSD), which incorporated access to local copies of NCBI databases and useful analysis tools, such as Clustal Omega and BLAST within one web service [42]. Unfortunately, it is not publicly available anymore.

### 2.5 Local Installation

An alternative to sequence data management via web services is represented by standalone versions of the above-mentioned tools or all-in-one software packages. Limitations concerning file upload and download rates of web services can be bypassed using this approach. However, it needs to be mentioned that using a conventional personal computer (PC) for the download and storage of large sequence data sets is not efficient, as the demands on computational power and storage are too high for such devices. Therefore, it is suggested to locate the management on servers or clouds for both data storage and analysis. Servers and clusters provide significantly more computational power and storage than any PC. Most companies, universities or other institutions provide their own server or even cluster environment. Clouds can be a possible alternative to apply efficient sequence data management. Data and software can be accessed and shared anytime and anywhere [43]. Researchers are able to work with growing sequence databases without the need to maintain computing facilities on their own. Cloud customers have the possibility to elastically grow and shrink their allocations depending on their current need for computational power and storage. However, cloud computing also shows some obstacles, like complicated rental processes, redesign of applications for parallel frameworks, time consuming transfers from and to clouds and higher costs [44].

#### 2.5.1 Data Retrieval

It needs to be mentioned that downloading and updating large amounts of data can be very time-consuming, require appropriate computational infrastructure, and knowledge of the data structure, content, and update cycles [43]. Adequate and well-organized data structures need to be established to work on big data sets. Databases and analysis environments can be set up manually or with the use of appropriate software packages. Unfortunately, none of the existing sequence management tools for local installation include automated data retrieval and filtering mechanisms.

A high number of sequence records can be retrieved by bulk downloads from the FTP servers of the mentioned data repositories. Local BLAST searches can be performed using the downloaded sequences and BLAST+, the command line application of BLAST. However, advanced computational knowledge is required for the implementation of a custom sequence database management system. For example, we experienced download times of several days for the download of all bacterial genomes from the NCBI FTP server. The uncompressed sequence records occupied over 1 Terabyte of storage on our server. Therefore, it is necessary to consider possible server time outs and enough free disc space to enable an efficient download process. Furthermore, the directory structures of the FTP servers need to be known, because different databases of NCBI show different folder structures. Whereas all sequence files of the GenBank division are located in one folder, whole genome sequences are divided by taxonomic ranks into different subdirectories. Additionally, these subdirectories do not comprise the actual sequence files, but a reference to it. Although NCBI provides helpful information concerning the download of genomic data from the FTP server, it still demands basic knowledge of command line applications and data structures to download sequence files. This knowledge is necessary to keep the downloaded data up to date, because of the exponential growth of sequence data (Fig. 3). Regular updates of local sequence databases are necessary to ensure an oligonucleotide design based on up to date data. Thus, well-defined update mechanisms need to be implemented to keep up with these growing data sets.

#### 2.5.2 Filtering & Analysis

Even more complex data management mechanisms need to be considered after data is downloaded. Working directly on the sequence files is impractical and needs a lot of memory, due to extensive read and write operations on sequence files that contain several Gigabytes of data. Each conventional text editor will fail during the attempt to open such files. Therefore, filtering demands the implementation of a suitable database model. Examples for possible standalone solutions for local data management tools, involving data structures, filtering and analysis, are provided by ARB and Mothur [45,46]. ARB is a software environment that enables the creation of custom sequence databases and applications of integrated and connected software tools for sequence analysis. Mothur users are able to analyze their community sequence data by just using one tool.

Besides these local data management packages, database systems can be set up manually to efficiently store sequence data. Here, the ever-growing amount of sequence data plays an important role in the selection of an adequate database system. For example, relational database models, such as MySQL [47] are usually not the first choice for saving large amounts of sequence data. Although, these database systems are very consistent and stable, their rigid structure is not suitable for sequence records that can show high differences in size and content. More flexible database models are needed to store large amounts of sequence data in meaningful data structures. One database system that proved successful with large amounts of sequence records is the document-based database model MongoDB [48]. It features a powerful database model for the creation of biological sequence databases and allows efficient interaction with the data, due to its high performance, scalability, and flexibility. Efficient data filtering can be accomplished as annotation, feature, and sequence data can be easily saved and retrieved using MongoDB. Another possibility to save sequence data within a data structure are graph databases. Their model consists of vertices that represent entities within the database, and edges, which are the relationship between two entities. For example, OrientDB [49] is an open source graph-based database management system written in Java. It provides a powerful database system, but basic knowledge concerning Java and the Java Runtime Environment is necessary to successfully use OrientDB for saving and updating sequence data.

Most of the previously mentioned tools for sequence similarity searches and alignments also provide standalone versions to run the algorithms locally on a server or on a cloud. Theoretically, unlimited analysis can be accomplished using these tools. For example, some of the command line applications of the alignment tools (BLAST+ and Clustal Omega) provide an option for multithreading. Thus, alignments are performed in parallel and a considerable speed-up of the analysis can be achieved. However, it needs to be assured that the hardware is capable of performing such an analysis.

#### 2.5.3 Limitations

Possible limitations for local installations of database management systems concern especially the computational knowledge of the users as well as hardware requirements. Setting up a local data management system requires at least basic knowledge of command line applications and database systems. Even the best hardware can be of no use if the data management system relies on poor implementations. No matter if data needs to be downloaded, updated or filtered, each data processing step needs to be well-implemented for optimal performance. The least inefficiency when working on big data sets can have drastic impacts on the performance as well as on the content of the databases. Additionally, adequate hardware is a must for working on big data sets. For example, BLAST searches on all bacterial genomes downloaded from NCBI require over 100 Gigabyte memory and optimally a high number of CPUs to run the analysis in parallel. Thus, hardware limitations have a direct impact on the performance and efficiency of a data management system.

## 3 Oligonucleotide Design

**Fig. 4:**
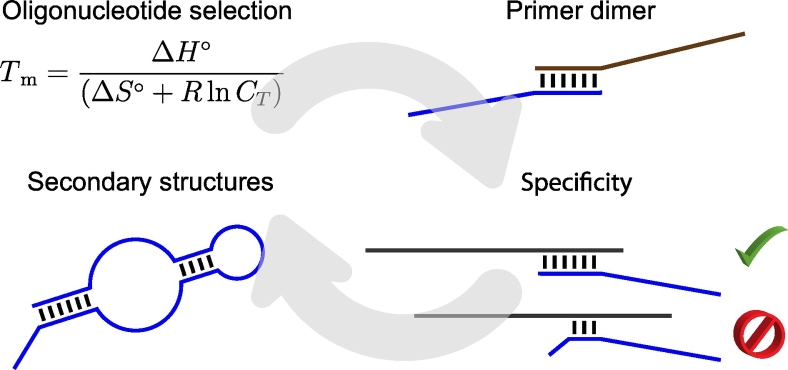
A simplification of the oligonucleotide design workflow. The most important steps are the oligonucleotide selection, primer dimer formation, secondary structure and specificity checks.

*In-silico* oligonucleotide design and optimization techniques are gaining importance for in-vitro applications [2]. The design of specific and sensitive assays is a challenging task because important key parameters need to be considered [50,51]. The following paragraphs describe these parameters in detail (see overview in Fig. 4). An overview of different applications for *in-silico* oligonucleotide design should give an insight into possible implementations of design paradigms. A tool should be well-chosen, as most of these tools differ in the number of covered key parameters during the design workflow.

### 3.1 Parameters

#### 3.1.1 Oligonucleotide Length

The length of an oligonucleotide is directly linked to its specificity, stability, efficiency and cost [1,51]. The longer an oligonucleotide, the higher its theoretical specificity due to its increasing uniqueness among targets. Each additional nucleotide increases the uniqueness of an oligonucleotide by a factor of four [1]. However, an increase in length can also lead to an increased nucleotide mismatch tolerance [51]. Therefore, long oligonucleotides tend to bind to more heterogeneous sequences. This is an advantage if a universal primer pair should amplify closely related gene families with similar sequences [1]. Further, the longer a primer or probe, the higher the possibility for hairpin formation or primer dimers (see paragraph *Primer dimers* below for further information). For example, PCR primers with a length of 18 and 24 base pairs (bp) tend to be specific if the annealing temperature (T_a_) of the reaction is adjusted to the melting temperature (T_m_) of the primer [1].

#### 3.1.2 Melting Temperature

The T_m_ is the predicted temperature at which 50% of the oligonucleotides are paired with their complementary sequence. In the worst case, erroneous estimations of the T_m_ can lead to no or unspecific binding [52]. The reaction temperature should be low enough to enable binding of the oligonucleotides to the target DNA. However, if the temperature is too low, unspecific binding or secondary structure formation might lead to a reduction of reaction efficiency [53]. Several factors, such as the structure of the oligonucleotides (e.g. their GC content), salt concentrations, and nearest neighbor (NN) interactions, need to be taken into account for the estimation of the T_m_ [54,55]. Several T_m_ estimation approaches were developed and used by oligonucleotide design software [54]. The most simple and earliest models just incorporated the GC content [56,57]. Howley et al. introduced a more sophisticated approach by including salt concentrations into the calculation [58]. More accurate prediction models of T_m_ were developed under the application of NN parameters, oligonucleotide and salt concentrations [[59], [60], [61], [62], [63], [64], [65], [66]]. Studies have shown that NN based T_m_ prediction models perform best [53,54,65,67].

Another considerable assumption is that the T_m_ of primer pairs should be in a close temperature range to ensure the same performance of both primers under the same experimental conditions [1,51,68]. However, SantaLucia et al. found some deficiencies within this assumption [69]. The authors argue that the hybridization behavior at a specific T_m_ is not equal to the one at the T_a_. Therefore, they suggest matching the Gibb's free energy change for the primer design rather than the T_m_ values.

#### 3.1.3 Specificity

It is important that oligonucleotides only bind to their intended target DNA. This implies the prevention of unspecific binding to other DNA, such as non-target DNA, and other regions within a template [70]. First, a well-defined set of target sequences based on reliable data management forms the basis for the design of specific oligonucleotides (see section 2 Data management). If these sequences are not chosen properly, the whole reaction might fail, because the sequences selected for the design do not match the sequences of the actual target DNA used in the reaction. Second, the oligonucleotides should not bind to other targets. One approach to check whether the oligonucleotides bind to unintended sequences is via sequence alignments. However, using just the sequence identity for a specificity check might be insufficient, as it does not cover DNA duplex thermodynamics [69]. The application of the NN model would be most efficient, as mismatches and matches contribute differently to the stability of a duplex (see paragraph *Secondary structure formation* for more information). Therefore, it is not recommended to only use BLAST for specificity checks, because thermodynamically important hybridization events, caused by stabilizing or destabilizing, are not discovered. Although mismatches can contribute to the stability of a duplex, mismatches at the 3′ end should be avoided for primer design, as they might lead to no amplification of the target sequences [51,71].

#### 3.1.4 Secondary Structure Formation

**Fig. 5:**
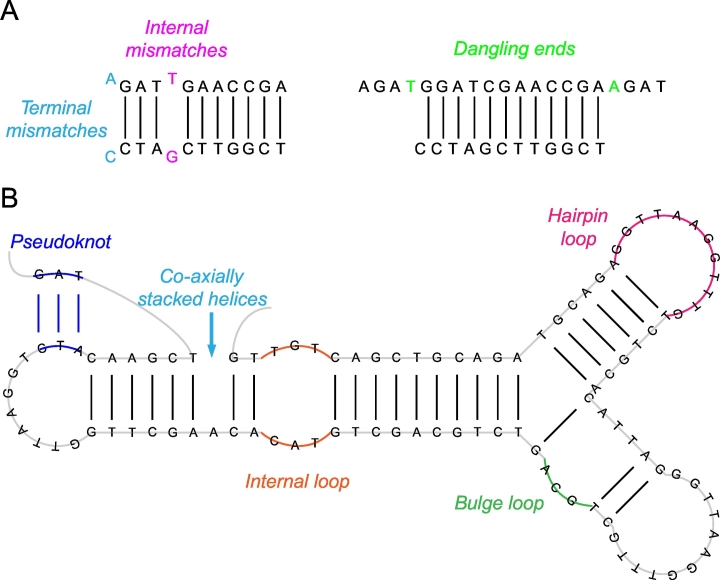
Simplified illustration of possible secondary structure formation considering dangling ends, terminal and internal mismatches, hairpin loops, bulge loops, internal loops, co-axially stacked helices and pseudoknots (modified, original figure by SantaLucia et al. [67]). The vertical black lines indicate base pairing between the nucleotides on both ends of the particular line. The secondary structure formation are colored.

As oligonucleotides are single-stranded DNA molecules, they can form secondary structures and limit the hybridization of oligonucleotides to the target DNA [[72], [73], [74], [75]]. SantaLucia and Hicks gave a comprehensive overview of the requirements for accurate DNA secondary structure predictions [67]. In their review, they emphasize to extend the NN model by salt dependence, loops, terminal dangling ends, and all possible internal and terminal mismatches [64,65,76,77]. The thermodynamic parameters for the NN model proposed by SantaLucia et al. were derived from multiple linear regression algorithms using sequences from a variety of labs and have been compared to other published NN parameters [65,67,78]. The inclusion of salt dependence enables an accurate prediction of thermodynamics for a wide range of buffer conditions within biological assays [69]. Dangling-end parameters (see Fig. 5 A) are of particular importance for PCR reactions, as the first unpaired bases next to both ends of the binding site of the primers have a great influence on the binding behavior [69]. It needs to be considered that mismatches are either stabilizing or destabilizing a duplex [67]. Internal and terminal mismatches (see Fig. 5 A) can have different influences on the stability of a duplex. Terminal mismatches need to be considered during secondary structure predictions, as the stability of a duplex strongly depends on the identity of a terminal mismatch, its closing Watson-Crick pair (meaning the Watson-Crick pair at the beginning and end of a duplex next to the terminal mismatch), and its orientation [77]. Except for terminal mismatches, thermodynamics of mismatches are independent of their position within a duplex.

Intramolecular folding, involving any kinds of loop motifs that occur in folded DNA, has a strong influence on the reaction behavior, as it can inhibit hybridization of oligonucleotides to their intended target DNA. Therefore, loop parameters for loop motifs, such as hairpin loops, bulge loops, internal loops, co-axially stacked helices, and multibranched loops (see Fig. 5 B), are important for secondary structure prediction and therefore also for primer and probe design [67].

#### 3.1.5 Primer Dimers

The hybridization of two primers to each other is referred to as primer dimer formation. Oligonucleotides can hybridize with other oligonucleotides, such as forward and reverse primers in PCR reactions or any kind of oligonucleotides in multiplex reactions. Secondary structure formation and primer dimers are two closely related phenomena. As for the secondary structure prediction, salt concentrations in combination with NN thermodynamics and additionally the total oligonucleotide strand concentration need to be involved in the prediction of primer dimer formation. It is important to explicitly consider primer dimers during oligonucleotide design, as they can lead to no or undesired binding. For example, if two primers hybridize at their 3′ ends, the DNA polymerase can still bind to the oligonucleotides and extend them in both directions generating an undesired product. If a primer binds to another one at the 5′ end or in the middle of the sequence, DNA polymerase cannot efficiently extend the primers [69]. In summary, primer dimer formation can significantly inhibit hybridization events in different assays and should be considered during the oligonucleotide design process.

### 3.2 Existing Tools

A wide range of different tools for oligonucleotide design is freely available. These tools show differences in their design workflows, especially concerning the implementation of the above-mentioned design parameters. None of the freely available tools takes all the key parameters fully into consideration for the design process. The reason is that trade-offs between the complexity of a prediction model and the computing time need to be made. A critical event of each oligonucleotide-based assay is the formation of specific binding between oligonucleotides and target DNA. However, these single-stranded polymers also tend to bind to unintended targets or themselves. The probability of such unspecific binding increases with the complexity of an assay. Accurate models need to be implemented to achieve an optimal abstraction of the actual reaction. The computational complexity of the simulation increases with the accuracy of a model predicting a reaction [2]. Furthermore, adequate hardware requirements need to be met for the implementation of complex simulation models. For example, the application of molecular dynamics simulations provides an accurate prediction of molecular interactions between oligonucleotides. One possibility to apply such simulation is by using oxDNA, a coarse-grained model to capture thermodynamic, structural, and mechanical characteristics of double- and single-stranded DNA [79]. However, the setup of such simulations requires accurate domain knowledge as well as non-negligible development time. Therefore, the development of an efficient oligonucleotide design workflow demands the right balance between the precision of the applied computer models, the general expenditure of time, and the computational workload. The time factor also includes the time for building a model as well as the computational run time of the workflow.

As already mentioned, a high number of oligonucleotide design tools is available online. With the highest respect to the authors, mentioning each tool would exceed the limits of this review. Therefore, we chose to discuss the tools which represent best the different implementations of *in-silico* oligonucleotide design. Further oligonucleotide design tools are listed in Supplemental Table S1.

#### 3.2.1 Singleplex Applications

Most of the available oligonucleotide design tools include basic design features, such as length, GC content, T_m_ of the oligonucleotide, as well as primer product sizes and salt concentrations of the reaction. However, differences concerning the implemented temperature prediction can arise and therefore lead to different results. A study comparing different T_m_ calculation software for PCR and RT-PCR has shown that Primer3 Plus and Primer-BLAST implement the most accurate T_m_ prediction models [53,80].

Primer3 is a helpful online, but also locally available, tool for singleplex oligonucleotide design. It incorporates up to date NN thermodynamic parameters and salt correction formulas for the prediction of secondary structures, hairpin formation as well as hybridization strength to the intended targets. However, no specificity check is applied by Primer3.

Specificity checks can be performed using Primer-BLAST. This tool provides the possibility to generate or check primer pairs, but not for probes. It implements the Primer3 algorithm for primer design and evaluation as well as BLAST along with the Needleman-Wunsch global alignment algorithm for the specificity check [81]. As a consequence, specificity checks of Primer-BLAST are only based on sequence similarity and do not include thermodynamically important hybridization events. Unfortunately, no local version of this tool is available. Therefore, specificity checks are limited to the reduced collection of databases provided by the web service.

#### 3.2.2 Multiplex Applications

Free multiplex applications are provided by MPprimer and oli2go [82,83]. MPprimer is a standalone tool that utilizes Primer3 to design five primer pair candidates by default for each DNA template. Next, the primers are checked for specificity and primer dimer formation. The specificity check is performed by an implementation of MFEprimer [84]. In this case, specificity checks are based on thermodynamics and not only sequence similarity. This software tool uses *k*-mer indexing and the NN model to evaluate the binding stability between primers and binding sites. However, the authors of MFEprimer only focus on the specificity concerning the 3′ ends of the primers. The non-target background DNA libraries are limited to a few model organisms, but additional organisms can be added upon request. Furthermore, the thermodynamic calculations do not involve the formation of dangling ends and intramolecular folding, like hairpins and loops. After specificity and dimerization checks are performed, a scoring matrix is generated based on these results. Primers without dimerization and nonspecific binding are scored with one and otherwise with zero. Finally, optimal primer pairs are chosen using a graph-expanding algorithm. However, it needs to be mentioned that a set of five primer pairs per template is relatively small. Though such a small number of primers will speed up the evaluation process, it is most likely that the final data set includes nonspecific binding or primer dimers, especially, if the assay involves many targets (e.g. >30 targets). These time savings lead to a reduction in quality.

In contrast, oli2go includes every possible, non-overlapping oligonucleotide candidate for a specificity check. This web tool is used for the design of primers and probes in multiplex reactions. It starts with the selection of all possible oligonucleotides based on length, T_m_ and hairpin threshold parameters. Possible probe candidates are afterwards checked for specificity using an implementation of BLAST+ and a user-defined selection of databases involving bacteria, viruses, plants, fungi, archaea, protozoa, and invertebrate. All possible probe candidates are taken into account for this specificity check. Although run time is affected by this high number of probes, the possibility of selecting specific oligonucleotides is increased. In contrast to MFEprimer, oli2go uses only sequence similarity for the specificity checks. The authors of oli2go argue this decision with the high number of sequences used for the specificity check. Indexing, like it is done in MFEprimer, is not possible due to the high amount of sequence data and the related memory usage. Therefore, BLAST has shown the best performance considering run time, memory usage, and result quality. After specific probes are selected, primers within the defined product size ranges are selected around the specific probes. The resulting primers are then checked for specificity against human DNA. All specific primers are finally checked for primer dimers regarding all relevant intermolecular structure formation. This primer dimer check is based on Primer3's thermodynamic alignment tool ntthal, which is based on the NN parameters presented by SantaLucia et al. [67].

A possible and freely available approach to include thermodynamics into specificity checks is provided by ThermoAlign [85]. This tool performs primer design for targeted resequencing with an option for multiplexing. Besides conventional primer design, T_m_ calculation based on the NN model, hairpin, and primer dimer prediction based on Primer3's ntthal tool, it performs thermodynamic alignments to check the specificity of the primer candidates. For this, primers are aligned to a reference genome using BLAST. Afterwards, the T_m_ of each off-target hit is estimated. Oligonucleotides that have a greater T_m_ than all predicted off-target binding sites are identified. In comparison to oli2go, ThermoAlign just uses one reference genome for the specificity check, whereas oli2go involves all available genomes from bacteria, viruses, plants, archaea, invertebrate, protozoa, and fungi. However, for such a high number of databases, it is not possible to perform thermoalignments within a reasonable run time at this moment.

One fee-based oligonucleotide design tool is Visual-OMP provided by DNA Software, Inc. This tool is based on the thermodynamic parameters suggested by SantaLucia et al. [67]. Visual-OMP gives users the possibility to design oligonucleotides for a variety of use cases, such as PCR, multiplex PCR, Taqman probes, microarrays, and molecular beacons. The software includes powerful design algorithms using the NN model and coupled multi-state equilibrium models to simulate thousands of possible oligonucleotide combinations. Additionally, DNA Software, Inc. provides a tool called ThermoBLAST to automatically scan oligonucleotides against large genome databases. This tool detects all thermodynamically stable hits including important mismatches.

### 3.3 Do it Yourself

Besides these existing tools, users can also generate their custom oligonucleotide design workflows. As for the data management, we suggest performing such calculations only on servers or clouds due to limitations of the computational infrastructure provided by conventional PCs. A possible example of a self-implemented oligonucleotide design workflow can be applied using command line applications provided by Primer3 and BLAST as well as workflow scripts that handle the different design steps. One possible script language is Python [86] as it includes a comprehensive library for biological data called Biopython [87]. Alternatively, Perl [88] and the R Project for Statistical Computing [89] also include packages for sequence handling and can be used to build primer design workflows. Furthermore, command line applications of Primer3 for T_m_, secondary structure and primer dimer predictions can be implemented. Databases, created during the data management step, can be used to generate BLAST databases to perform specificity checks using BLAST+.

However, especially primer dimer and specificity checks require enough memory and CPUs. A high number of CPU cores allow time-consuming design steps to be performed in parallel.

## 4 Summary

In summary, the efficient *in-silico* design of DNA oligonucleotides is gaining importance in the field of molecular biology. Generating specific and sensitive assays via software tools saves a lot of time and costs when it comes to in-vitro evaluation. However, the increasing amount of biological data and the complexity of simulation models for the design of oligonucleotides are challenging factors for automated workflows. A set of comprehensive and updated target and non-target sequences are essential to optimize the specificity of oligonucleotides. Furthermore, algorithms that simulate specific reactions, such as primer dimer formation, need to achieve the right balance between the precision of the applied computer models, the general expenditure of time, and computational workload.

## Funding

This work was supported by the European Union's Horizon 2020 research and innovation program [634137]. Funding for open access charge: H2020 [634137].

## Appendix A Supplementary Data

Supplementary material Image 1

## References

[1] Dieffenbach C., Lowe T., Dveksler G. (1993). General concepts for PCR primer design. Genome Res.

[2] Royce R., Santalucia J., Hicks D. (2003). Building an in silico laboratory for genomic assay design. Pharm Visions.

[3] Noguera D.R., Wright E.S., Camejo P., Yilmaz L.S. (2014). Mathematical tools to optimize the design of oligonucleotide probes and primers. Appl Microbiol Biotechnol.

[4] Park D.J. (2011). PCR protocols.

[5] Domingues L. (2017). PCR: methods and protocols.

[6] Jia Y., Conn P.M. (2013). Real-Time PCR. Laboratory methods in cell biology, Vol. 112 of methods in cell biology.

[7] Jebbink J., Bai X., Rogers B.B., Dawson D.B., Scheuermann R.H., Domiati-Saad R. (2003). Development of real-time PCR assays for the quantitative detection of epstein-barr virus and cytomegalovirus, comparison of taqman probes, and molecular beacons. J Mol Diagn.

[8] Chandler L. (2013). Sources of errors in molecular testing. Accurate results in the clinical laboratory.

[9] Bumgarner R. (2013). Overview of DNA microarrays: types, applications, and their future, current protocols in molecular biology chapter 22.

[10] Vradi L., Luo J.L., Hibbs D.E., Perry J.D., Anderson R.J., Orenga S. (2017). Methods for the detection and identification of pathogenic bacteria: past, present, and future. Chem Soc Rev.

[11] Krizkova S., Kepinska M., Emri G., Rodrigo M.A.M., Tmejova K., Nerudova D. (2016). Microarray analysis of metallothioneins in human diseases - a review. J Pharm Biomed Anal.

[12] Ko J., Park S.-G., Lee S., Wang X., Mun C., Kim S. (2018). Culture-free detection of bacterial pathogens on plasmonic nanopillar arrays using rapid Raman mapping. ACS Appl Mater Interfaces.

[13] Frickmann H., Zautner A.E., Moter A., Kikhney J., Hagen R.M., Stender H. (2017). Fluorescence in situ hybridization (FISH) in the microbiological diagnostic routine laboratory: a review. Crit Rev Microbiol.

[14] Miller C.M., Harris E.N. (2016). Antisense oligonucleotides: treatment strategies and cellular internalization. RNA Dis. (Houston, Tex.).

[15] INSDC http://www.insdc.org/.

[16] NCBI https://www.ncbi.nlm.nih.gov/.

[17] DDBJ https://www.ddbj.nig.ac.jp.

[18] EMBL https://www.ebi.ac.uk/.

[19] Mashima J., Kodama Y., Fujisawa T., Katayama T., Okuda Y., Kaminuma E. (2016). DNA data bank of Japan. Nucleic Acids Res.

[20] Coordinators N.R. (2015). Database resources of the national center for biotechnology information. Nucleic Acids Res.

[21] Brooksbank C., Bergman M.T., Apweiler R., Birney E., Thornton J. (2013). The european bioinformatics institutes data resources 2014. Nucleic Acids Res.

[22] ENA https://www.ebi.ac.uk/ena.

[23] Quast C., Pruesse E., Yilmaz P., Gerken J., Schweer T., Yarza P. (2012). The SILVA ribosomal RNA gene database project: improved data processing and web-based tools. Nucleic Acids Res.

[24] Jia B., Raphenya A.R., Alcock B., Waglechner N., Guo P., Tsang K.K. (2016). CARD 2017: expansion and model-centric curation of the comprehensive antibiotic resistance database. Nucleic Acids Res.

[25] Chowdhury B., Garai G. (2017). A review on multiple sequence alignment from the perspective of genetic algorithm. Genomics.

[26] Rehm B. (2001). Bioinformatic tools for DNA/protein sequence analysis, functional assignment of genes and protein classification. Appl Microbiol Biotechnol.

[27] Sievers F., Higgins D.G. (2018). Clustal omega for making accurate alignments of many protein sequences. Protein Sci.

[28] Lassmann T., Sonnhammer E.L. (2005). Kalign-an accurate and fast multiple sequence alignment algorithm. BMC bioinforma.

[29] Edgar R.C. (2004). MUSCLE: multiple sequence alignment with high accuracy and high throughput. Nucleic Acids Res.

[30] Katoh K., Standley D.M. (2013). MAFFT multiple sequence alignment software version 7: improvements in performance and usability. Mol Biol Evol.

[31] Notredame C., Higgins D.G., Heringa J. (2000). T-coffee: a novel method for fast and accurate multiple sequence alignment. J Mol Biol.

[32] Al Ait L., Yamak Z., Morgenstern B. (2013). DIALIGN at GOBICSmultiple sequence alignment using various sources of external information. Nucleic Acids Res.

[33] Altschul S.F., Gish W., Miller W., Myers E.W., Lipman D.J. (1990). Basic local alignment search tool. J Mol Biol.

[34] Sayers E.W., Cavanaugh M., Clark K., Ostell J., Pruitt K.D., Karsch-Mizrachi I. (2018). GenBank. Nucleic Acids Res.

[35] Park Y.M., Squizzato S., Buso N., Gur T., Lopez R. (2017). The EBI search engine: EBI search as a servicemaking biological data accessible for all. Nucleic Acids Res.

[36] Pruitt K.D., Tatusova T., Maglott D.R. (2005). NCBI reference sequence (RefSeq): a curated non-redundant sequence database of genomes, transcripts and proteins. Nucleic Acids Res.

[37] Ye J., Coulouris G., Zaretskaya I., Cutcutache I., Rozen S., Madden T.L. (2012). Primer-BLAST: a tool to design target-specific primers for polymerase chain reaction. BMC Bioinforma.

[38] Li W., Godzik A. (2006). Cd-hit: a fast program for clustering and comparing large sets of protein or nucleotide sequences. Bioinformatics.

[39] Edgar R.C. (2010). Search and clustering orders of magnitude faster than BLAST. Bioinformatics.

[40] Haeussler M., Zweig A.S., Tyner C., Speir M.L., Rosenbloom K.R., Raney B.J. (2018). The UCSC genome browser database: 2019 update. Nucleic Acids Res.

[41] Afgan E., Baker D., Batut B., Van Den Beek M., Bouvier D., Čech M. (2018). The galaxy platform for accessible, reproducible and collaborative biomedical analyses: 2018 update. Nucleic Acids Res.

[42] Givan S.A., Sullivan C.M., Carrington J.C. (2007). The personal sequence database: a suite of tools to create and maintain web-accessible sequence databases. BMC bioinforma.

[43] Marx V. (2013). Biology: the big challenges of big data. Nature.

[44] Schatz M.C., Langmead B., Salzberg S.L. (2010). Cloud computing and the DNA data race. Nat Biotechnol.

[45] Ludwig W., Strunk O., Westram R., Richter L., Meier H., Yadhukumar (2004). ARB: a software environment for sequence data. Nucleic Acids Res.

[46] Schloss P.D., Westcott S.L., Ryabin T., Hall J.R., Hartmann M., Hollister E.B. (2009). Introducing mothur: open-source, platform-independent, community-supported software for describing and comparing microbial communities. Appl Environ Microbiol.

[47] MySQL https://www.mysql.com/.

[48] MongoDB https://www.mongodb.com/.

[49] OrientDB https://www.orientdb.com/.

[50] Kämpke T., Kieninger M., Mecklenburg M. (2001). Efficient primer design algorithms. Bioinformatics.

[51] Burpo F.J. (2001). A critical review of PCR primer design algorithms and crosshybridization case study. Biochemistry.

[52] Steger G. (1994). Thermal denaturation of double-stranded nucleic acids: prediction of temperatures critical for gradient gel electrophoresis and polymerase chain reaction. Nucleic Acids Res.

[53] Bakhtiarizadeh M.R., Najaf-Panah M.J., Mousapour H., Salami S.A. (2016). Versatility of different melting temperature (Tm) calculator software for robust PCR and real-time PCR oligonucleotide design: a practical guide. Gene Rep.

[54] Chavali S., Mahajan A., Tabassum R., Maiti S., Bharadwaj D. (2005). Oligonucleotide properties determination and primer designing: a critical examination of predictions. Bioinformatics.

[55] Herrmann M.G., Dobrowolski S.F., Wittwer C.T. (2000). Rapid β-globin genotyping by multiplexing probe melting temperature and color. Clin Chem.

[56] Marmur J., Doty P. (1962). Determination of the base composition of deoxyribonucleic acid from its thermal denaturation temperature. J Mol Biol.

[57] Wallace R.B., Shaffer J., Murphy R., Bonner J., Hirose T., Itakura K. (1979). Hybridization of synthetic oligodeoxyribonucleotides to X 174 DNA: the effect of single base pair mismatch. Nucleic Acids Res.

[58] Howley P.M., Israel M.A., Law M.-F., Martin M.A. (1979). A rapid method for detecting and mapping homology between heterologous DNAs. Evaluation of polyomavirus genomes. J Biol Chem.

[59] Breslauer K.J., Frank R., Blöcker H., Marky L.A. (1986). Predicting DNA duplex stability from the base sequence. Proc Natl Acad Sci.

[60] Freier S.M., Kierzek R., Jaeger J.A., Sugimoto N., Caruthers M.H., Neilson T. (1986). Improved free-energy parameters for predictions of RNA duplex stability. Proc Natl Acad Sci.

[61] Sugimoto N., Nakano S.-i., Katoh M., Matsumura A., Nakamuta H., Ohmichi T. (1995). Thermodynamic parameters to predict stability of RNA/DNA hybrid duplexes. Biochemistry.

[62] Sugimoto N., Nakano S.-i., Yoneyama M., Honda K.-i. (1996). Improved thermodynamic parameters and helix initiation factor to predict stability of DNA duplexes. Nucleic Acids Res.

[63] SantaLucia J., Allawi H.T., Seneviratne P.A. (1996). Improved nearest-neighbor parameters for predicting DNA duplex stability. Biochemistry.

[64] Allawi H.T., SantaLucia J. (1997). Thermodynamics and NMR of internal G T mismatches in DNA. Biochemistry.

[65] SantaLucia J. (1998). A unified view of polymer, dumbbell, and oligonucleotide DNA nearest-neighbor thermodynamics. Proc Natl Acad Sci.

[66] Xia T., SantaLucia J., Burkard M.E., Kierzek R., Schroeder S.J., Jiao X. (1998). Thermodynamic parameters for an expanded nearest-neighbor model for formation of RNA duplexes with Watson-Crick base pairs. Biochemistry.

[67] SantaLucia J., Hicks D. (2004). The thermodynamics of DNA structural motifs. Annu Rev Biophys Biomol Struct.

[68] Schretter C., Milinkovitch M.C. (2005). Oligonucleotide design by multilevel optimization.

[69] SantaLucia J. (2007). Physical principles and visual-OMP software for optimal PCR design. PCR primer design.

[70] Rychlik W., Rhoads R.E. (1989). A computer program for choosing optimal oligonucleotides for filter hybridization, sequencing and in vitro amplification of DNA. Nucleic Acids Res.

[71] Kwok S., Kellogg D., McKinney N., Spasic D., Goda L., Levenson C. (1990). Effects of primer-template mismatches on the polymerase chain reaction: human immunodeficiency virus type 1 model studies. Nucleic Acids Res.

[72] Stull R.A., Taylor L.A., Szoka F.C. (1992). Predicting antisense oligonucleotide inhibitory efficacy: a computational approach using histograms and thermodynamic indices. Nucleic Acids Res.

[73] Mathews D.H., Burkard M.E., Freier S.M., Wyatt J.R., Turner D.H. (1999). Predicting oligonucleotide affinity to nucleic acid targets. RNA.

[74] Nazarenko I., Pires R., Lowe B., Obaidy M., Rashtchian A. (2002). Effect of primary and secondary structure of oligodeoxyribonucleotides on the fluorescent properties of conjugated dyes. Nucleic Acids Res.

[75] Okumoto Y., Ohmichi T., Sugimoto N. (2002). Immobilized small deoxyribozyme to distinguish RNA secondary structures. Biochemistry.

[76] Bommarito S., Peyret N., Jr J.S. (2000). Thermodynamic parameters for DNA sequences with dangling ends. Nucleic Acids Res.

[77] Peyret N., Seneviratne P.A., Allawi H.T., SantaLucia J. (1999). Nearest-neighbor thermodynamics and NMR of DNA sequences with internal A A, C C, G G, and T T Mismatches. Biochemistry.

[78] Owczarzy R., Vallone P.M., Gallo F.J., Paner T.M., Lane M.J., Benight A.S. (1997). Predicting sequence-dependent melting stability of short duplex DNA oligomers, biopolymers. Original Res Biomol.

[79] Snodin B.E., Randisi F., Mosayebi M., Šulc P., Schreck J.S., Romano F. (2015). Introducing improved structural properties and salt dependence into a coarse-grained model of DNA. J Chem Phys.

[80] Untergasser A., Cutcutache I., Koressaar T., Ye J., Faircloth B.C., Remm M. (2012). Primer3 - new capabilities and interfaces. Nucleic Acids Res.

[81] Needleman S.B., Wunsch C.D. (1970). A general method applicable to the search for similarities in the amino acid sequence of two proteins. J Mol Biol.

[82] Shen Z., Qu W., Wang W., Lu Y., Wu Y., Li Z. (2010). MPprimer: a program for reliable multiplex PCR primer design. BMC bioinforma.

[83] Hendling M., Pabinger S., Peters K., Wolff N., Conzemius R., Barišić I. (2018). Oli2go: an automated multiplex oligonucleotide design tool. Nucleic Acids Res.

[84] Qu W., Zhou Y., Zhang Y., Lu Y., Wang X., Zhao D. (2012). MFEprimer-2.0: a fast thermodynamics-based program for checking PCR primer specificity. Nucleic Acids Res.

[85] Francis F., Dumas M.D., Wisser R.J. (2017). ThermoAlign: a genome-aware primer design tool for tiled amplicon resequencing. Sci Rep.

[86] Python https://www.python.org/.

[87] Cock P.J., Antao T., Chang J.T., Chapman B.A., Cox C.J., Dalke A. (2009). Biopython: freely available Python tools for computational molecular biology and bioinformatics. Bioinformatics.

[88] Perl https://www.perl.org/.

[89] R https://www.r-project.org/.

