# Supplementary material for: In-silico Design of DNA Oligonucleotides: Challenges and Approaches
Source: Comput Struct Biotechnol J. 2019 Jul 29;17:1056–65. doi: 10.1016/j.csbj.2019.07.008 (PMC6700205; doi:10.1016/j.csbj.2019.07.008)
Supplement: Supplementary file 1 — Supplementary material [file mmc1.docx]

**Supplementary Material**

**Table S1. A selection of oligonucleotide design tools.**

| Tool | Primer Design | Probe Design | Hairpin Check | Batch design* | Probe Specificity Check | Primer Specificity Check | Cross Dimer Check | Multiplexing |
| --- | --- | --- | --- | --- | --- | --- | --- | --- |
| Oli2go (1) | Yes | Yes | Yes | Yes | Yes | Yes | Yes | Yes |
| MSP-HTPrimer (2) | Yes | Yes | Yes | Yes | No | No | No | No |
| PrecisePrimer (3) | Yes | No | No | Yes | No | No | No | No |
| Primer3 (4) | Yes | Yes | Yes | No | No | No | No | No |
| MFEprimer (5) | No | No | Yes | Yes | Yes | Yes | Yes | Yes |
| Primer-BLAST (6) | Yes | No | No | No | No | Yes | Yes | No |
| AutoPrime (7) | Yes | Yes | Yes | No | No | No | No | No |
| EvOligo (8) | Yes | Yes | Yes | Yes | No | No | Yes | Yes |
| OLIGO (9) | Yes | Yes | Yes | Yes | No | No | Yes | Yes |
| QuantPrime (10) | Yes | No | No | No | No | Yes | No | No |
| GenScript (11) | Yes | Yes | Yes | No | No | No | No | No |
| RExPrimer (12) | Yes | No | Yes | No | No | No | No | No |
| BatchPrimer3 (13) | Yes | Yes | Yes | Yes | No | No | Yes | Yes |
| ThermoAlign (14) | Yes | Yes | Yes | Yes | Yes | Yes | Yes | Yes |

* Batch design means the application of the software on more than one input sequences in one run. However, this term does not cover multiplexing, as design steps such as specificity and secondary structure checks need to be performed for multiplex applications.

**References**

1. Hendling, Michaela, et al. Oli2go: an automated multiplex oligonucleotide design tool. *Nucleic acids research.* 2018, 46, S. W252-W256.

2. Pandey, Ram Vinay, et al. MSP-HTPrimer: a high-throughput primer design tool to improve assay design for DNA methylation analysis in epigenetics. *Clinical epigenetics.* 2016, 8, S. 101.

3. Pauthenier, Cyrille und Faulon, Jean-Loup. PrecisePrimer: an easy-to-use web server for designing PCR primers for DNA library cloning and DNA shuffling. *Nucleic acids research.* 2014, 42, S. W205-W209.

4. Untergasser, Andreas, et al. Primer3 - new capabilities and interfaces. *Nucleic acids research.* 2012, 40.

5. Qu, Wubin, et al. MFEprimer-2.0: a fast thermodynamics-based program for checking PCR primer specificity. *Nucleic acids research.* 2012, 40, S. W205-W208.

6. Ye, Jian, et al. Primer-BLAST: a tool to design target-specific primers for polymerase chain reaction. *BMC Bioinformatics.* 2012, 13, S. 134.

7. Wrobel, Gunnar, Kokocinski, Felix und Lichter, Peter. AutoPrime: selecting primers for expressed sequences. *Genome Biology.* 2004, 5, S. P11.

8. Milewski, Marek C, et al. EvOligo: A Novel Software to Design and Group Libraries of Oligonucleotides Applicable for Nucleic Acid-Based Experiments. *Journal of Computational Biology.* 2017, 24, S. 1014-1028.

9. Rychlik, W. OLIGO 7 primer analysis software. [Buchverf.] A Yuryev. *Methods in Molecular Biology.* s.l. : Humana Press, 2007, Bd. 402.

10. Arvidsson, Samuel, et al. QuantPrime – a flexible tool for reliable high-throughput primer design for quantitative PCR. *BMC Bioinformatics.* 2008, 9, S. 465.

11. GenScript Online PCR Primers Designs Tool. [Online] https://www.genscript.com/tools/pcr-primers-designer.

12. Piriyapongsa, Jittima, et al. RExPrimer: an integrated primer designing tool increases PCR effectiveness by avoiding 3'SNP-in-primer and mis-priming from structural variation. *BMC Genomics.* 2009, 10, S. S4.

13. You, Frank M, et al. BatchPrimer3: a high throughput web application for PCR and sequencing primer design. *BMC Bioinformatics.* 2008, 9, S. 253.

14. Francis, Felix, Dumas, Michael D und Wisser, Randall J. ThermoAlign: a genome-aware primer design tool for tiled amplicon resequencing. *Scientific Reports.* 2017, 7, S. 44437.
